# Supplementary material for: MMP2 Polymorphism Affects Plasma Matrix Metalloproteinase (MMP)-2 Levels, and Correlates with the Decline in Lung Function in Hypersensitivity Pneumonitis Positive to Autoantibodies Patients
Source: Biomolecules. 2019 Oct 5;9(10):574. doi: 10.3390/biom9100574 (PMC6843910; doi:10.3390/biom9100574)
Supplement: Supplementary file 1 [file biomolecules-09-00574-s001.pdf]

Supplementary Materials: The following are available online at www.mdpi.com/xxx/s1, Table S1: title,

Supplementary Table 1. SNPs and associated alleles in the genes *MMP1* and *MMP2* in patients with hypersensitivity pneumonitis versus hypersensitivity pneumonitis with positive autoantibodies.

Gene/SNP	AA	HPAbs+ *vs.* HP		HPAbs+ *vs.* HC		HP *vs.* HC	
		p-value	OR (CI 95%)	p-value	OR (CI 95%)	p-value	OR (CI 95%)
MMP1 rs7125062	T	0.4	1.29 (0.74-2.23)	<0.001	3.69 (2.16-6.29)	<0.001	2.97 (1.99-4.09)
MMP2 rs11646643	A	0.03	1.88 (1.06-3.33)	0.01	2.35 (1.36-4.05)	0.2	1.25 (0.88-1.75)

*p-values are presented after Bonferroni's correction. AA: Allele associated; HPAbs+: HP patients with autoantibodies positive; HP: hypersensitivity pneumonitis patients without autoantibodies; HC: healthy controls.

Supplementary Table 2. Frequencies of alleles and genotypes in the *MMP1* gene (collagenase) in patients with hypersensitivity pneumonitis and hypersensitivity pneumonitis with positive autoantibodies.

**Gen/SNP**	**HPAbs+ n=34**		**HP n=104**		**HC n=184**		**HapMap-MEX***
	**n**	**AF/GF (%)**	**n**	**AF/GF (%)**	**n**	**AF/GF (%)**	**AF/GF (%) n=100**
MMP1							
rs470215							
A	56	82.35	170	81.73	306	83.15	79.00
G	12	17.65	38	18.27	62	16.85	21.00
AA	23	67.65	69	66.35	129	70.11	59.70
AG	10	29.41	32	30.77	48	26.09	39.00
GG	1	2.94	3	2.88	7	3.80	1.30
rs7125062							
C	30	44.12	105	50.48	274	74.45	47.00
T	38	55.88	103	49.52	94	25.55	53.00
CC	7	20.59	27	25.96	135	73.37	18.00
CT	16	47.06	51	49.04	4	2.17	58.00
TT	11	32.35	26	25.00	45	24.46	24.00
rs2071232							
T	53	77.94	145	69.71	273	74.18	74.00
C	15	22.06	63	30.29	95	25.82	26.00
TT	19	55.88	49	47.12	99	53.80	54.00
CT	15	44.12	47	45.19	75	40.76	40.00
CC	0	0.00	8	7.69	10	5.43	6.00

HPAbs+: HP patients with autoantibodies positive; HP: hypersensitivity pneumonitis patients without autoantibodies; HC: Healthy controls; AF: Allele frequency; GF: Genotype frequency. *HapMap-MEX: Populational data from 100 subjects with Mexican ancestry residing in Los Angeles CA, USA.

Supplementary Table 3. Frequencies of alleles and genotypes in the genes *MMP2* and *MMP9* (gelatinases) in patients with hypersensitivity pneumonitis and hypersensitivity pneumonitis with positive autoantibodies.

Gen/SNP	HPAbs+ n=34		HP n=104		HC n=184		HapMap-MEX*
	n	AF/GF (%)	n	AF/GF (%)	n	AF/GF (%)	AF/GF(%) n=100
MMP2							
rs243839							
A	42	61.76	140	67.31	264	71.74	73.00
G	26	38.24	68	32.69	104	28.26	27.00
AA	12	35.29	45	43.27	87	47.28	54.00
AG	18	52.94	50	48.08	90	48.91	38.00
GG	4	11.76	9	8.65	7	3.80	8.00
rs243835							
T	33	48.53	103	49.52	214	58.15	40.00
C	35	51.47	105	50.48	154	41.85	60.00
TT	9	26.47	23	22.12	62	33.70	18.00
TC	15	44.12	57	54.81	90	48.91	44.00
CC	10	29.41	24	23.08	32	17.39	38.00
rs243864							
T	54	79.41	172	82.69	288	78.26	78.00
G	14	20.59	36	17.31	80	21.74	22.00
TT	22	64.71	71	68.27	112	60.87	58.00
TG	10	29.41	30	28.85	64	34.78	40.00
GG	2	5.88	3	2.88	8	4.35	2.00
rs11646643							
G	23	33.82	102	49.04	201	54.62	39.00
A	45	66.18	106	50.96	167	45.38	61.00
GG	2	5.88	35	33.65	77	41.85	8.00
GA	19	55.88	32	30.77	47	25.54	62.00
AA	13	38.24	37	35.58	60	32.61	30.00
MMP9							
rs3918253							
C	58	85.29	188	90.38	320	86.96	78.00
T	10	14.71	20	9.62	48	13.04	22.00
CC	24	70.59	85	81.73	145	78.80	64.50
CT	10	29.41	18	17.31	30	16.30	25.00
TT	0	0.00	1	0.96	9	4.89	10.50
rs3918278							
G	68	100	206	99.04	364	98.91	94.00
A	0	0.00	2	0.96	4	1.09	6.00
GG	34	100	103	99.04	180	97.83	89.60
GA	0	0.00	0	0.00	4	2.17	9.50
AA	0	0.00	1	0.96	0	0.00	0.90

HPAbs+: HP patients with autoantibodies positive; HP: hypersensitivity pneumonitis patients without autoantibodies; HC: Healthy controls; AF: Allele frequency; GF: Genotype frequency. *HapMap-MEX: Populational data from 100 subjects with Mexican ancestry residing in Los Angeles CA, USA.

Supplementary Table 4. Frequencies of alleles and genotypes in the *MMP12* gene (metalloelastase) in patients with hypersensitivity pneumonitis and hypersensitivity pneumonitis with positive autoantibodies.

Gen/SNP	HPAbs+ n=34		HP n=104		HC n=184		HapMap-MEX*
	n	AF/GF (%)	n	AF/GF (%)	n	AF/GF (%)	AF/GF (%) n=100
MMP12							
rs12808148							
T	63	92.65	195	93.75	350	95.11	92.00
C	5	7.35	13	6.25	18	4.89	8.00
TT	29	85.29	91	87.50	167	90.76	84.00
TC	5	14.71	13	12.50	16	8.70	16.0
CC	0	0.00	0	0.00	1	0.54	0.00
rs17368659							
G	65	95.59	198	95.19	357	97.01	91.0
T	3	4.41	10	4.81	11	2.99	9.0
GG	31	91.18	94	90.38	174	94.57	84.0
GT	3	8.82	10	9.62	9	4.89	14.0
TT	0	0.00	0	0.00	1	0.54	2.0
rs2276109							
A	65	95.59	200	96.15	358	97.28	91.0
G	3	4.41	8	3.85	10	2.72	9.0
AA	31	91.18	96	92.31	175	95.11	84.0
AG	3	8.82	8	7.69	8	4.35	14.0
GG	0	0.00	0	0.00	1	0.54	2.0

HPAbs+: HP patients with autoantibodies positive; HP: hypersensitivity pneumonitis patients without autoantibodies; HC: Healthy controls; AF: Allele frequency; GF: Genotype frequency. *HapMap-MEX: Populational data from 100 subjects with Mexican ancestry residing in Los Angeles CA, USA.

Supplementary Table 6. SNPs and associated genotypes in the genes *MMP1* and *MMP2* in patients with hypersensitivity.

Gene/ Model	SNP/ Genotype	HPAbs+ *vs.* HP		HPAbs+ *vs.* HC		HP *vs.* HC		HP (all) *vs.* HC	
		p	OR (CI 95%)	p	OR (CI 95%)	p	OR (CI 95%)	p	OR (CI 95%)
MMP1	rs7125062								
Codominant	CC		1		1		1		1
	CT	0.3	1.21 (0.44–3.30)	0.0002	77.1 (20.3–292.6)	<0.001	63.75 (21.25–191.20)	<0.001	66.51 (22.66-195.18)
	TT		1.63 (0.54–4.85)		4.7 (1.7–12.8)		2.8 (1.53–5.45)		3.26 (1.84-5.80)
Dominant	CC								
	CT+TT	0.64	1.35 (0.52-3.46)	<0.001	10.62 (4.34-25.96)	<0.001	7.85 (4.54-13.57)	<0.001	8. 42 (5.07-13.98)
MMP2	rs11646643								
Codominant	GG		1		1		1		1
	GA	0.04	10.39 (2.2-48.1)	0.007	15.56 (3.46–69.85)	0.2	1.49 (0.82–2.73)	0.013	2.26 1.29-3.94
	AA		6.14 (1.2–29.2)		8.34 (1.81–38.38)		1.35 (0.76–2.40)		1.73 1.01-2.98
Dominant	GG								
	GA+AA	0.001	8.11 (1.83-35.84)	<0.001	11.51 (2.67-49.49)	0.2	1.41 (0.85-2.34)	0.006	1.96 (1.21-3.16)

HPAbs+: HP patients with autoantibodies positive; HP: hypersensitivity pneumonitis patients without autoantibodies; HC: healthy controls.

Supplementary Table 7. SNPs and genotypes in the gen *MMP9* in patients with hypersensitivity pneumonitis.

Gene/Model	SNP/ Genotype	Genotype frequency (%)			HPAbs+ *vs*. HP		HPAbs+ *vs*. HC		HP *vs*. HC	
		HPAbs+ (n=34)	HP (n=104)	HC (n=184)	p	OR (CI 95%)	p	OR (CI 95%)	p	OR (CI 95%)
MMP9	rs3918253									
Codominant	CC	70.59	81.73	78.80	0.28	1	0.10	1	0.22	1
	CT	29.41	17.31	16.30		1.97 (0.80-4.82)		2.01 (0.87-4.64)		1.02 (0.53-1.95)
	TT	0.00	0.96	4.89		0.0 (---)		0.0 (---)		0.19 (0.02-1.52)
Dominant	CC	70.59	81.73	78.80						
	CT+TT	29.41	18.27	21.20	0.22	1.86 (0.76-4.53)	0.37	1. 54 (0.68-3. 51)	0.64	0.83 (0.45-1.52)
	rs3918278									
Codominant	GG	100.00	99.04	97.83	---	1	---	1	0.13	1
	GA	0.00	0.00	2.17		NV		NV		0.00
	AA	0.00	0.96	0.00		NV		NV		---
Dominant	GG	100.00	99.04	97.83						
	GA+AA	0.00	0.96	2.17		NV		NV	0.65	0.43 (0.04-3.96)

HPAbs+: HP patients with autoantibodies positive; HP: hypersensitivity pneumonitis patients without autoantibodies; HC: healthy controls; NV: null value.

Supplementary Table 8. SNPs and genotypes in the gene *MMP9* and in patients with hypersensitivity pneumonitis.

Gene/Model	SNP/ Genotype	Genotype frequency (%)			HPAbs+ *vs.* HP		HPAbs+ *vs.* HC		HP vs. HC	
		HPAbs+ (n=34)	HP (n=104)	HC (n=184)	p	OR (CI 95%)	p	OR (CI 95%)	p	OR (CI 95%)
MMP12	rs12808148									
Codominant	TT	85.29	87.50	90.76		1		1		1
	TC	14.71	12.50	8.70	---	NV	0.50	1.79 (0.61-5.29)	0.45	1.49 (0.68-3.24)
	CC	0.00	0.00	0.54		NV		NV		NV
Dominant	TT	85.29	87.50	90.76						
	TC+CC	14.71	12.50	9.24	0.77	1.12 (0.39-3.67)	0.35	1.69 (0.57-4.94)	0.42	1.4 (0.65-3.01)
	rs17368659									
Codominant	GG	91.18	90.38	94.57		1		1		1
	GT	8.82	9.62	4.89	—	NV	0.60	1.87 (0.48-7.29)	0.15	2.30 (0.90-5.87)
	TT	0.00	0.00	0.54		NV		NV		NV
Dominant	GG	91.18	90.38	94.57						
	GT+TT	8.82	9.62	5.43	0.84	0.9 (0.23- 3.51)	0.43	1.68 (0.43-6.46)	0.22	1.85 (0.74-4.60)
	rs2276109									
Codominant	AA	91.18	92.31	95. 11		1		1		1
	AG	8.82	7.69	4.35	—	NV	0.50	2.11 (0.53-8.42)	0.37	1.82 (0.66-5.01)
	GG	0.00	0.00	0.54		NV		NV		NV
Dominant	AA	91.18	92.31	95.11						
	AG+GG	8.82	7.69	4.89	0.87	1.16 (0.29-4.65)	0.4	1.88 (0.48-7.34)	0.43	1.62 (0.60-4.33)

HPAbs+: HP patients with autoantibodies positive; HP: hypersensitivity pneumonitis patients without autoantibodies; HC: healthy controls. NV: null value.
